# Leveraging Nursing Assessment for Early Identification of Post Operative Gastrointestinal Dysfunction (POGD) in Patients Undergoing Colorectal Surgery

**DOI:** 10.3390/curroncol31070276

**Published:** 2024-06-29

**Authors:** Tessy Siby, Alice Shajimon, Daniel Mullen, Shahnaz Gillani, Jeffrey R. Ong, Nikki E. Dinkins, Brittany Kruse, Carla Patel, Craig Messick, Nicole Gourmelon, Mary R. Butler, Vijaya Gottumukkala

**Affiliations:** 1 Clinical Nursing, Division of Nursing, The University of Texas MD Anderson Cancer Center, Houston, TX 77030, USA; tsiby@mdanderson.org (T.S.); ashajim@mdanderson.org (A.S.); dmullen@mdanderson.org (D.M.); smgillani@mdanderson.org (S.G.); 2 Clinical & Access Applications, The University of Texas MD Anderson Cancer Center, Houston, TX 77030, USA; jrong1@mdanderson.org (J.R.O.); ndinkins@mdanderson.org (N.E.D.); 3 Nursing Administration, Division of Nursing, The University of Texas at MD Anderson Cancer Center, Houston, TX 77030, USA; bccampbell@mdanderson.org (B.K.); carbaker@mdanderson.org (C.P.); 4 Colon & Rectal Surgery, Division of Surgery, The University of Texas at MD Anderson Cancer Center, Houston, TX 77030, USA; cmessick@mdanderson.org (C.M.); ncgourmelon@mdanderson.org (N.G.); 5 Nursing Clinical Informatics, The University of Texas at MD Anderson Cancer Center, Houston, TX 77030, USA; mbutler@mdanderson.org; 6 Department of Anesthesiology & Perioperative Medicine, The University of Texas MD Anderson Cancer Center, Houston, TX 77030, USA

**Keywords:** colorectal surgery, postoperative gastrointestinal dysfunction, nursing education, patient outcomes

## Abstract

*Background:* Postoperative gastrointestinal dysfunction (POGD) remains a common morbidity after gastrointestinal surgery. POGD is associated with delayed hospital recovery, increased length of stay, poor patient satisfaction and experience, and increased economic hardship. The I-FEED scoring system was created by a group of experts to address the lack of a consistent objective definition of POGD. However, the I-FEED tool needs clinical validation before it can be adopted into clinical practice. The scope of this phase 1 Quality Improvement initiative involves the feasibility of implementing percussion into the nursing workflow without additional burden. *Methods:* All gastrointestinal/colorectal surgical unit registered nurses underwent comprehensive training in abdominal percussion. This involved understanding the technique, its application in postoperative gastrointestinal dysfunction assessment, and its integration into the existing nursing documentation in the Electronic Health Record (EHR). After six months of education and practice, a six-question survey was sent to all inpatient GI surgical unit nurses about incorporating the percussion assessment into their routine workflow and documentation. *Results:* Responses were received from 91% of day-shift nurses and 76% of night-shift registered nurses. Overall, 95% of the nurses were confident in completing the abdominal percussion during their daily assessment. *Conclusion:* Nurses’ effective use of the I-FEED tool may help improve patient outcomes after surgery. The tool could also be an effective instrument for the early identification of postoperative gastrointestinal dysfunction (POGD) in surgical patients.

## 1. Introduction

**Figure 1:**
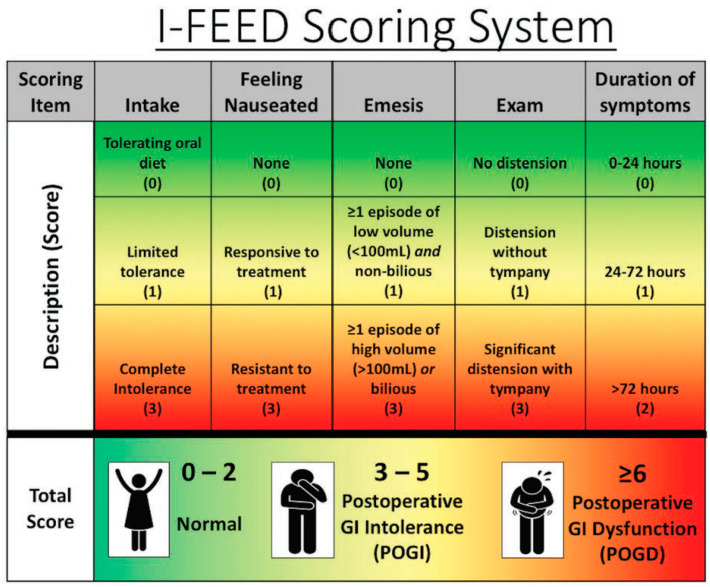
The I-FEED scoring system was created out of the need for a consistent objective GI function. The scoring system attributes based on the clinical presentation of the patients and categorizes patients into normal (0–2), postoperative GI intolerance (3–5), and postoperative GI dysfunction (≥6). GI, gastrointestinal; I-FEED, **I**ntake, **F**eeling nauseated, **E**mesis physical **E**xam, and **D**uration of symptoms; POGD, postoperative gastrointestinal dysfunction; POGI, postoperative gastrointestinal intolerance. Reprinted from Ref. [3].

**Figure 2:**
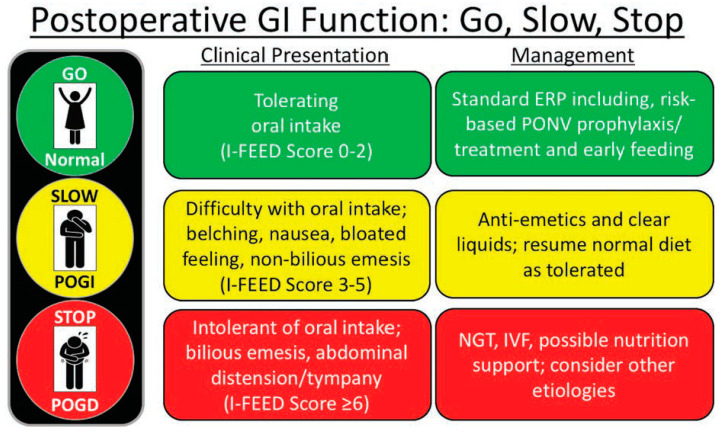
A treatment algorithm was developed based on the I-FEED scoring system for the management of patients with impaired postoperative GI function according to the clinical presentation of the patient in real time. ERP, enhanced recovery protocol; GI, gastrointestinal; I-FEED, Intake, **F**eeling nauseated, **E**mesis physical **E**xam, and **D**uration of symptoms; IVF, intravenous fluids; BGT, nasogastric tube; POGD, postoperative gastrointestinal dysfunction; POGI, postoperative gastrointestinal intolerance; PONV, postoperative nausea and vomiting. Reprinted from Ref. [3].

MD Anderson Cancer Center is a comprehensive cancer referral and academic cancer hospital with a 32-bed gastrointestinal surgical unit (GISU). Many of the patients admitted to the GISU would have had open or minimal access to colorectal and abdominal surgeries within an enhanced recovery pathway (ERP). ERPs focus on avoiding overnight fasting, allowing clear liquids until two hours before surgery, multimodal opioid-sparing pain management strategies, rational (optimal) fluid therapy avoiding undue fluid restriction or fluid overload, early diet advancement, early ambulation, and aggressive bowel management principles. The main goal of an ERP is to minimize symptom burden, minimize postoperative complications, enhance functional recovery, and reduce the length of hospital stay [1]. Postoperative ileus (POI) occurs in up to 30% of patients after colorectal surgery, which causes increased morbidity, prolonged length of stay, and higher cost of care [2]. The American Society for Enhanced Recovery and Perioperative Quality Initiative (ASER POQI) group developed the I-FEED scoring system as a valuable tool to evaluate and manage POI and POGD objectively [3]. The I-FEED tool assesses the patients’ intake, nausea, emesis, abdominal exam, and duration of symptoms, allowing for early identification of POGD (Figure 1). While the I-FEED tool is not currently validated and widely adopted into clinical practice, it has been proposed as a valuable tool in the management of POGD (Figure 2).

Figure 1 and Figure 2 show the I-FEED tool and the proposed treatment algorithm for managing postoperative gastrointestinal dysfunction, respectively (Courtesy of Hedrick et al., 2018 [3], with permission from Dr. Timothy E. Miller, MB, ChB, FRCA, for the Perioperative Quality Initiative (POQI) 2 Workgroup).

## 2. Available Knowledge

Traditionally, delayed GI recovery was considered a common and unavoidable consequence of surgery. However, delayed GI recovery is no longer inevitable due to the proliferation of enhanced recovery pathways. Enhanced recovery pathways (ERPs) strive to minimize the psychological and physiological stress response to major surgeries [4]. Minimizing POGD after colorectal surgery continues to be a significant challenge for clinicians. The current literature is ambiguous regarding the definition of postoperative GI dysfunction (POGD). A validated tool to identify the early symptoms of POGD would be invaluable in managing POGD [5]. 

## 3. Material and Methods

This Quality Improvement Project was approved by the MD Anderson Quality Improvement Board and is included in the MD Anderson’s Quality Improvement Project Registry.

Before this initiative, the standard practice was for frontline nurses to perform a complete head-to-toe assessment at the beginning of the shift. If a patient experiences abdominal distention, nausea, or vomiting, the nurse would reassess the patient and notify the provider (without any advanced abdominal assessment) for further management. The GI surgery unit nurse’s abdominal assessment focused on abdominal inspection and auscultation for bowel sounds.

The implementation phase consisted of compiling didactic education material, including a video presentation on proper percussion of a patient by the GI surgical unit educator (File S1). The educator checked off the unit super users, and the super users helped to check off all other nursing staff. The education was completed using a PowerPoint presentation and videos on the techniques for proper percussion assessment. The check-off for accurate percussion assessment was achieved for all clinical nurses in the gastrointestinal colorectal surgical unit. All nurses were checked off for the skill of either an actual patient or in simulation. Newly hired nurses who joined the team after initial education were educated and checked off during their clinical orientation either by the nurse educator or their clinical preceptor.

## 4. Statistical Analysis

The Raosoft sample size calculator determined that a sample size of 377 assessments would give a 5% margin error, 95% confidence level, and 50% response distribution. Data were collected between March 2022 and February 2023 and were separated into two six-month periods: the first six months (n = 209) and the second six months (n = 168).

## 5. Data Collection and Results

After education and practice over six months, a six-question survey was sent to all inpatient GI surgical unit nurses about incorporating the percussion assessment into their routine workflow and documentation (QR code for the Qualtrics Survey—File S2). The survey had questions about how comfortable the nurses felt performing the percussion assessment and whether adding the percussion assessment to the head-to-toe assessment was non-burdensome and easy. A total of 91% of day-shift registered nurses (21/23) and 76% (13/17) of night-shift registered nurses responded.

Regarding their experience in the GISU, 15% reported less than one year of experience, 35% of nurses had worked for 1-5 years, and 50% had more than five years of experience. When asked if they had any previous experience performing percussion assessments before the education was completed for the unit, 12% reported not having any prior understanding, 53% had some experience, and 32% had good knowledge of performing percussion assessments. When asked how confident the nurses felt about performing the percussion assessment, 35% reported being somewhat comfortable, while 62% reported being very satisfied in performing and documenting the percussion assessment. When asked if it was easy to incorporate the percussion assessment into the head-to-toe evaluation, 12% reported that it was not easy, and 85% reported that it was feasible to incorporate the percussion assessment. When asked about the time it took to perform the abdominal percussion, 56% reported less than one minute, 38% said more than one minute, and only 6% reported that it takes more than 2 minutes to perform abdominal percussion for their patients (Table 1).

## 6. Implications for Nursing

The primary purpose of this exercise was to leverage a thorough abdominal nursing assessment and documentation for the early identification of POGD. Gaining confidence in performing the new assessment can take time for nurses. However, with education and training, most nurses agreed that incorporating abdominal percussion into their routine shift assessment of patients was feasible. As part of this ongoing initiative, the team will conduct a prospective exercise to assess the congruency of findings between nursing and provider exams; then, the team will study the utility of this tool in the management of POGD.

## 7. Discussion

Abdominal assessment, including percussion, is primary education for all nurses; however, advanced abdominal assessment (tympany) is not required in many hospitals as part of the head-to-toe assessment. In the gastrointestinal surgical unit, adding the advanced abdominal assessment, which includes percussion, to the nursing shift assessment could identify POGD early. This would allow prompt intervention for POGD, minimize patient suffering, improve patient experience, decrease hospital length of stay, and impact the cost of care. Nurses on the gastrointestinal surgical floors were educated on abdominal percussion, and following the training, 95% of the nurses were confident in completing abdominal percussion during their daily assessment. An additional row was added under the gastrointestinal flow sheet for percussion (dull/tympany) in the EHR for the nurses’ assessment documentation. As part of the new nurse orientation, abdominal percussion assessments were included. While incorporating abdominal percussion in nursing shift assessment, the nurses can potentially identify early symptoms and signs of POGD and communicate with the provider early, preventing further complications related to POGD.

## 8. Limitations

This is an education and implementation exercise of a single institution quality improvement project, and therefore, the findings are relevant to our institutional practice. While reviewing the data, it was noticed that there was a difference in the assessment and documentation among the nurses and providers. This implies that continued collaboration should occur between our nurses, nurse educators, and providers. Efforts are underway to continue educating nurses and providers on the tool’s utility.

Another essential consideration and limitation is the documentation in the EHR. Under abdominal inspection, options included rounded and distended, which could be interchangeable with large girth and distended abdomen. Having unified definitions is essential for accurate data entry.

Finally, we did not measure any changes or improvements in clinical outcomes as a direct result of this study.

## 9. Conclusions

In conclusion, the I-FEED tool could be an effective instrument for the early identification of POGD in gastrointestinal surgery patients. The results of our ongoing quality improvement initiatives on the congruency of examination findings between the nurses and providers and the evaluation of the clinical utility of the I-FEED tool in the algorithmic management of POGD will be forthcoming.

## Figures and Tables

**Table 1:** Post-education survey results.

Variable		Percentage
Years of experience in GI surgical oncology	Less than one year	15%
	Between 1 and 5 years	35%
	More than five years	50%
Previous experience performing abdominal percussion assessment	No experience before education	12%
	Some experience before education	53%
	Adequate knowledge of percussion	32%
Feeling confident performing abdominal percussion	Somewhat confident	35%
	Very confident	62%
How challenging was it to incorporate the abdominal percussion assessment	It is not feasible to include a percussion assessment	12%
	Feasible to incorporate percussion assessment	85%
Amount of time it takes to perform the percussion assessment	Less than 1 minute	56%
	More than 1 minute	38%
	Over 2 minutes	6 %
Total survey responses received	Day-shift nurse responses	91%
	Night-shift nurse responses	76%

## Data Availability

Relevant data pertaining to this project have been submitted as Supplementary Materials.

## Supplementary Materials

The following supporting information can be downloaded at https://www.mdpi.com/article/10.3390/curroncol31070276/s1, File S1: Educational material; File S2: Survey.
