# Supplementary material for: Leveraging Nursing Assessment for Early Identification of Post Operative Gastrointestinal Dysfunction (POGD) in Patients Undergoing Colorectal Surgery
Source: Curr Oncol. 2024 Jun 29;31(7):3752–7. doi: 10.3390/curroncol31070276 (PMC11276471; doi:10.3390/curroncol31070276)
Supplement: Supplementary file 1 [file curroncol-31-00276-s001.zip › File S1 iFEED Tool- Nursing Education.pdf]

I-FEED ASSESSMENT TOOL

- **Objectives:**

- Define I-FEED Assessment tool
- Discuss the rationale and expectations
- Discuss the benefits for using it for the enhanced recovery pathway

- Developed by Shahnaz Gillani
- Guidance from Nicole Gourmelon (PA) and Dr. Messick Craig

Introduction

- "American Society for Enhanced Recovery and Perioperative Quality Initiative Joint Consensus Statement on Postoperative Gastrointestinal Dysfunction Within an Enhanced Recovery Pathway for Elective Colorectal Surgery" (2017 International Anesthesia Research Society).
- Postoperative ileus is common complication & is associated with significant morbidity and cost.
- Recovery of GI functions after elective surgery is key to ***Enhanced Recovery Pathway***.

- **Common terms used in I-FEED assessment**
- **POI:** Postoperative ileus
- **POGI:** Postoperative Gastrointestinal Intolerance
- **POGD:** Postoperative Gastrointestinal Dysfunction
- **I-FEED:** Intake, feeling nauseated, emesis, examination, duration of symptoms

Limits for I-FEED Scores

- **I-FEED Score limits:**
- Normal 0-2
- POGI 3-5
- POGD 6+ points

- When documentation done for each element, the scores will automatically get calculated.

Change in Practice

- Once a shift assessment of the components of I-FEED (intolerance, feeling nauseated, emesis, exam, duration of symptoms).

- **Recommendations: to be done with the head to toe assessment at the beginning of the shift. Also, if the patient's condition has a change (e.g. emesis at 1400) then you would assess and document again.**

- I-FEED documentation tool is being developed in OneConnect and would soon be available.
- All nurses will be checked off for percussion by the educator, CNLs & charge nurses, once this presentation has been reviewed including the YouTube videos X2.

Percussion

- Definition: Striking or tapping of the surface of a part of the body for diagnostic or therapeutic purposes.
- Percussion is part of abdominal assessment especially for patients post-operative Gastro-Intestinal surgeries.
- Please watch the **first 30 seconds** of both YouTube videos for the correct percussion technique.

- https://youtu.be/OAUZRMGH1RY and https://youtu.be/lEJXQ-JmEr4

What are you looking for when percussing the abdomen

- Proper percussion technique: Expose the abdomen (don't do over the hospital gown for best results) and flattening out the bed. Use one finger to tap the distal joint of the middle finger, striking firmly, using the wrist to balance.

- Concept behind percussion: air-filled areas will be tympanic (like a drum), and solid/fluid filled areas sound duller.

- Defining possible results: tympany, resonance, dullness. We are mostly focused on what tympany is for purposes of GI dysfunction.

- Abdominal percussion (general assessment only, no need for assessing organomegaly or shifting dullness, etc).

Difference between Tympany & Dull on Percussion

Tympany Sound on Percussion

- Percussion of the abdomen results in a hollow, or drum-like sound
- This finding may be present in one, many or all of the abdomen
- Represents the distention of the bowels (stomach, small or large intestine) with air
- May reflect ileus, delayed return of bowel function, or impending flatus, or bowel function
- Typically noticed when the abdomen is distended.
- Still appreciated in obese abdomens

Dull Sound on Percussion

- Percussion results in lack of a hollow, or drum-like sound
- Typically occurs when the abdomen is not distended.
- Still appreciated in obese abdomens.
- When dull sounds noticed in one place, it is typically throughout the entire abdomen.

Evaluating Gastrointestinal Functional Recovery

iFEED

I-FEED Scoring System

Scoring Item	Intake	Feeling Nauseated	Emesis	Exam	Duration of symptoms
Description (Score)	Tolerating oral diet (0)	None (0)	None (0)	No distension (0)	0-24 hours (0)
	Limited tolerance (1)	Responsive to treatment (1)	≥1 episode of low volume (<100mL) *and* non-bilious (1)	Distension without tympany (1)	24-72 hours (1)
	Complete Intolerance (3)	Resistant to treatment (3)	≥1 episode of high volume (>100mL) *or* bilious (3)	Significant distension with tympany (3)	>72 hours (2)
Total Score	0 – 2 Normal		3 – 5 Postoperative GI Intolerance (POGI)		≥6 Postoperative GI Dysfunction (POGD)

Anesth Analg 2018; 126: 1896–907

THE UNIVERSITY OF TEXAS
MD Anderson Cancer Center
The Institute for Cancer Care Innovation

Evaluating Gastrointestinal Functional Recovery

iFEED

Postoperative GI Function: Go, Slow, Stop

	Clinical Presentation	Management
GO Normal	Tolerating oral intake (I-FEED Score 0-2)	Standard ERP including, risk-based PONV prophylaxis/ treatment and early feeding
SLOW POGI	Difficulty with oral intake; belching, nausea, bloated feeling, non-bilious emesis (I-FEED Score 3-5)	Anti-emetics and clear liquids; resume normal diet as tolerated
STOP POGD	Intolerant of oral intake; bilious emesis, abdominal distension/tympany (I-FEED Score ≥6)	NGT, IVF, possible nutrition support; consider other etiologies

Anesth Analg 2018; 126: 1896–907

Assessment Flowsheet Template – Abdomen Inspection

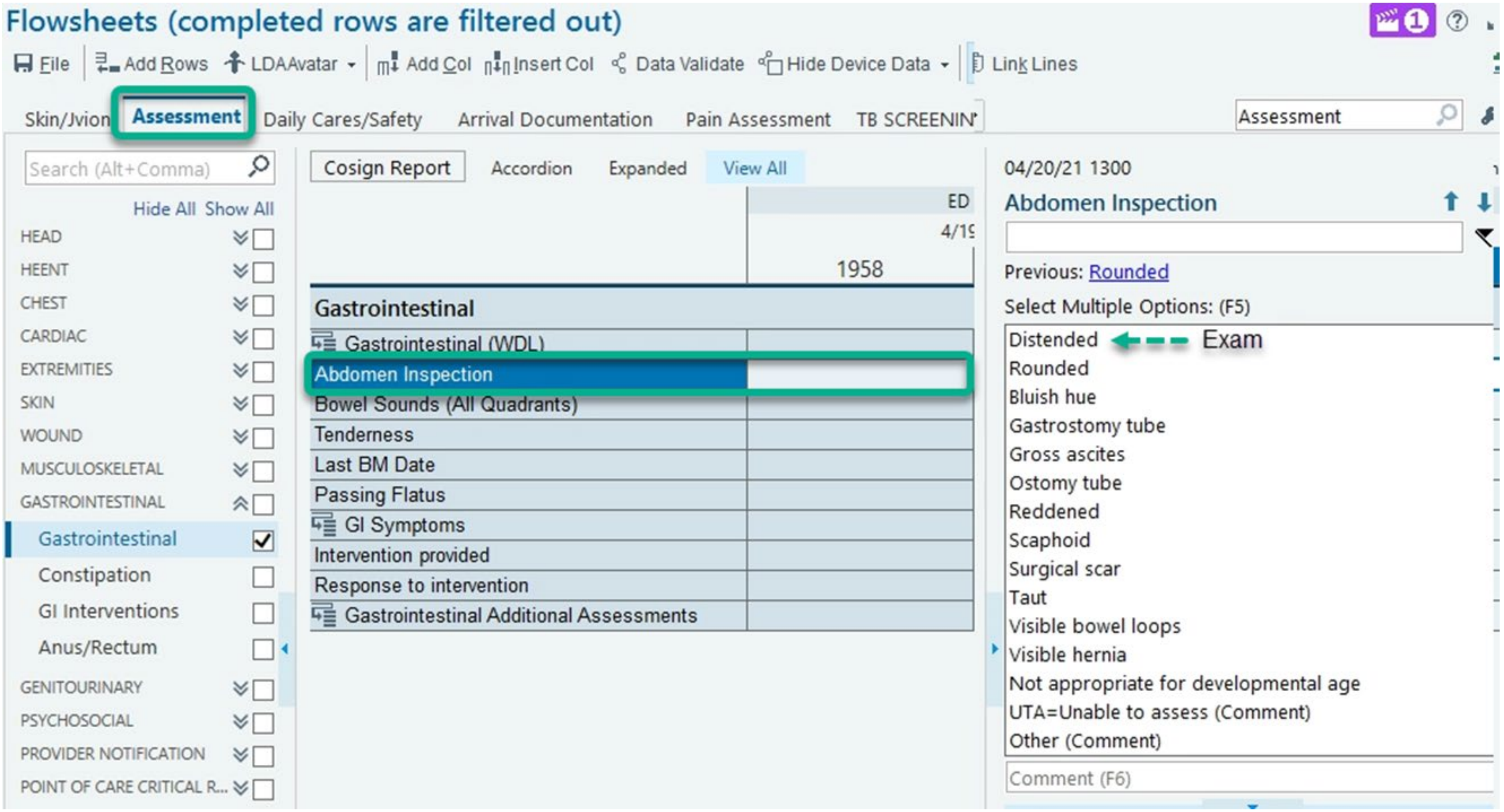

THE UNIVERSITY OF TEXAS
MD Anderson Cancer Center
The Institute for Cancer Care Innovation

Assessment Flowsheet Template – GI Symptoms

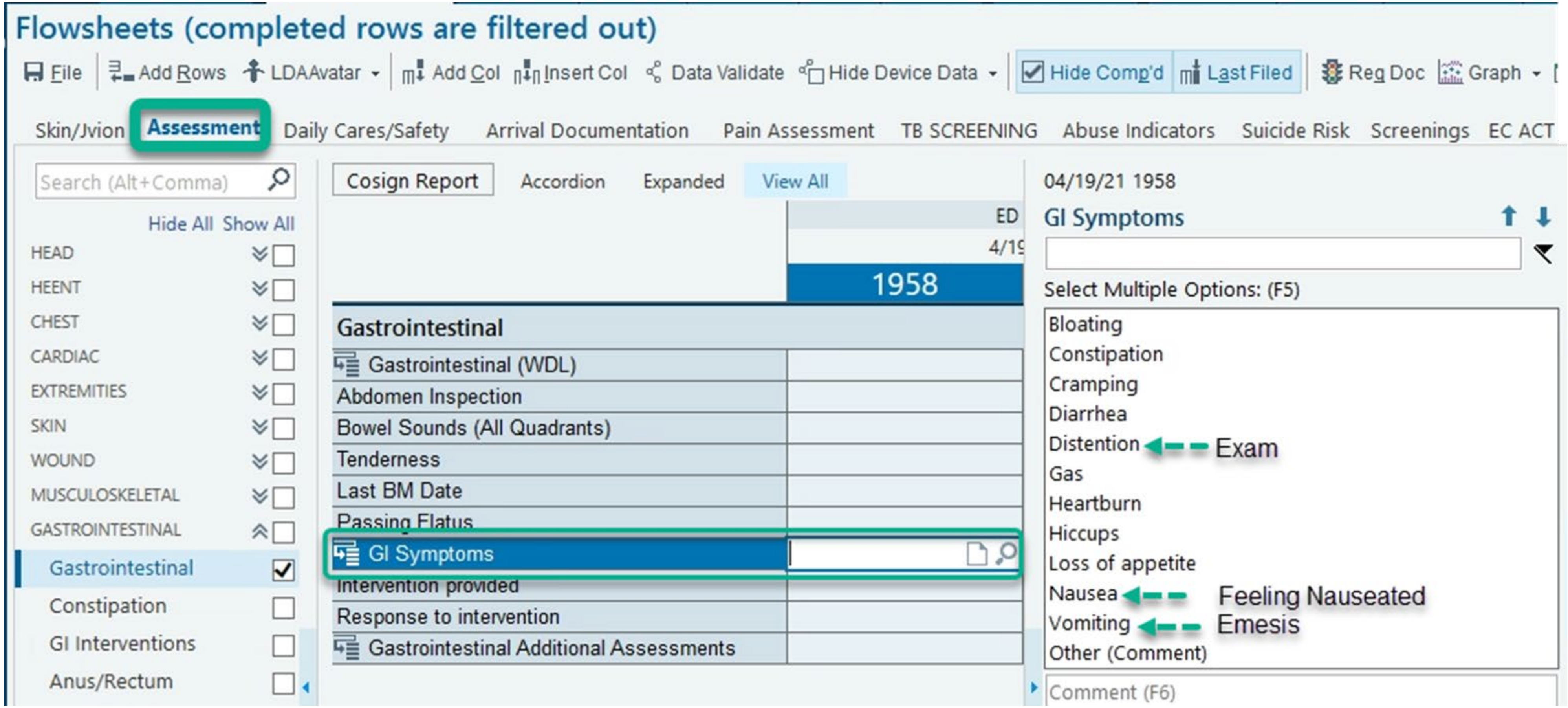

THE UNIVERSITY OF TEXAS
MD Anderson Cancer Center
The Institute for Cancer Care Innovation

Intake/Output Flowsheet Template – Percent Meals Eaten (%)

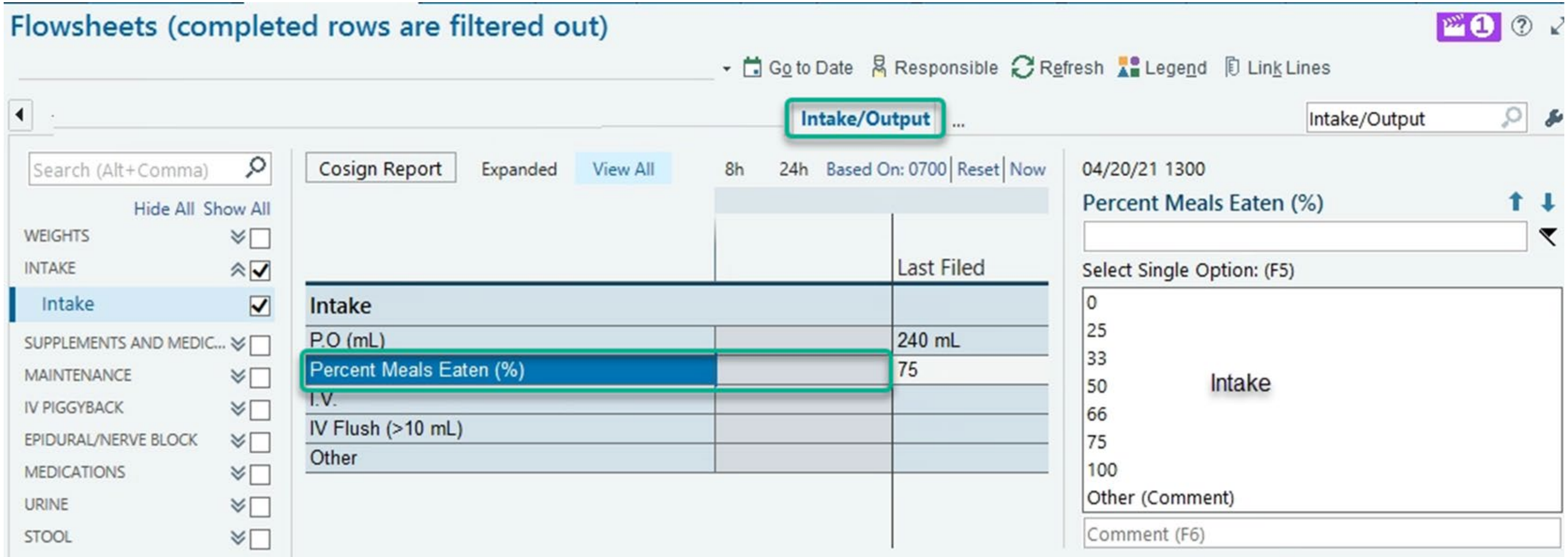

Intake/Output Flowsheet Template – Emesis (mL) – Emesis Amount

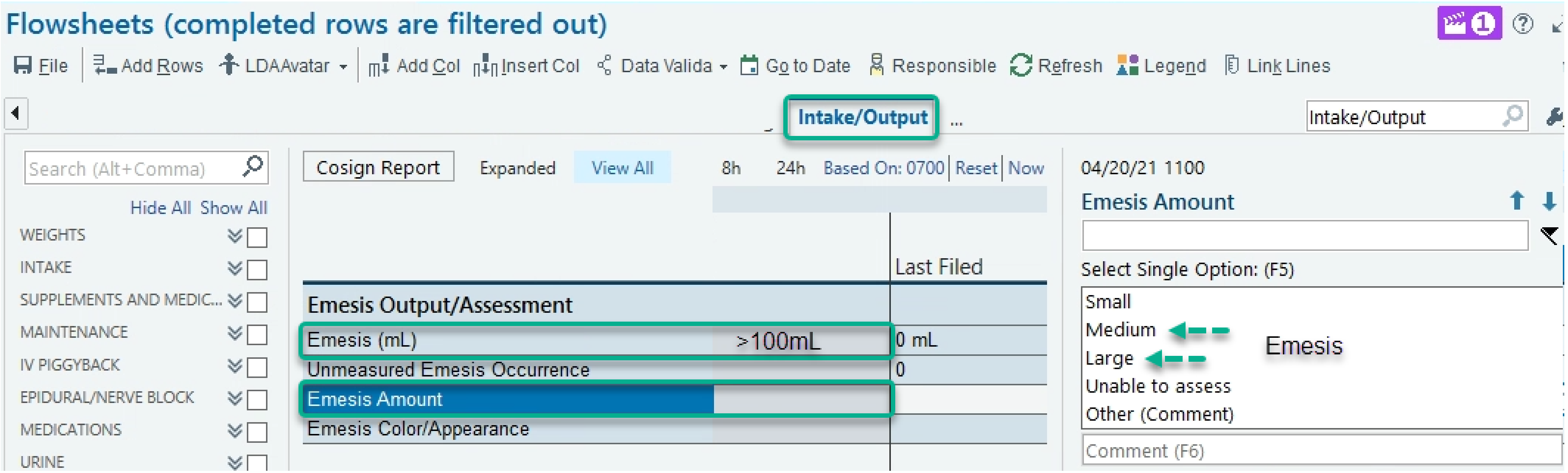

THE UNIVERSITY OF TEXAS MD Anderson Cancer Center
The Institute for Cancer Care Innovation

Intake/Output Flowsheet Template – Emesis Color/Appearance

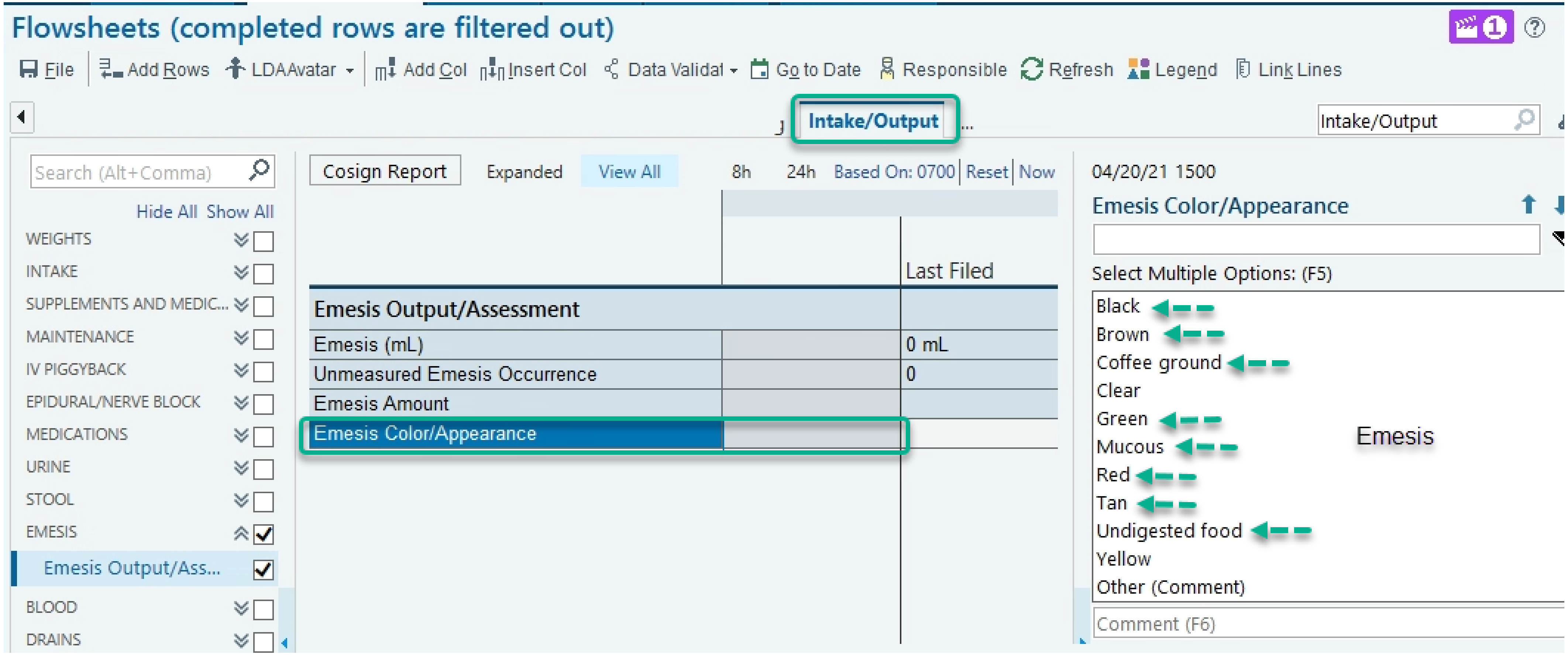

THE UNIVERSITY OF TEXAS MD Anderson Cancer Center
The Institute for Cancer Care Innovation

Questions

- For questions, please reach out to your educator- Shahnaz Gillani by email smgillani@mdanderson.org

- You will also have a chance to ask questions when we check you off.
