# Supplementary figures and images for: Leveraging Nursing Assessment for Early Identification of Post Operative Gastrointestinal Dysfunction (POGD) in Patients Undergoing Colorectal Surgery
Source: Curr Oncol. 2024 Jun 29;31(7):3752–7. doi: 10.3390/curroncol31070276 (PMC11276471; doi:10.3390/curroncol31070276)

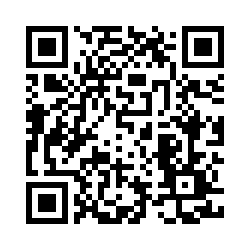

Supplement: Supplementary file 1 [file curroncol-31-00276-s001.zip › File S2 iFEED Tool-QR code- Qualtrics Survey.png]
